# Silencing YY1 Alleviates Ox-LDL-Induced Inflammation and Lipid Accumulation in Macrophages through Regulation of PCSK9/ LDLR Signaling

**DOI:** 10.4014/jmb.2207.07011

**Published:** 2022-09-23

**Authors:** Zhengyao Qian, Jianping Zhao

**Affiliations:** The Second Department of Cardiology, Tianjin Hospital, No.406, Jiefang South Road, Hexi District, Tianjin 300211, P.R. China

**Keywords:** Macrophage, lipid accumulation, cholesterol metabolism, inflammatory response, YY1

## Abstract

The formation of macrophage foam cells stimulated by oxidized low-density lipoprotein (ox-LDL) is deemed an important cause of atherosclerosis. Transcription factor Yin Yang 1 (YY1), which is a universally expressed multifunctional protein, is closely related to cell metabolism disorders such as lipid metabolism, sugar metabolism, and bile acid metabolism. However, whether YY1 is involved in macrophage inflammation and lipid accumulation still remains unknown. After mouse macrophage cell line RAW264.7 cells were induced by ox-LDL, YY1 and proprotein convertase subtilisin/kexin type 9 (PCSK9) expressions were found to be increased while low-density lipoprotein receptor (LDLR) expression was lowly expressed. Subsequently, through reverse transcription‐quantitative polymerase chain reaction (RT-qPCR), Western blot analysis, Oil Red O staining and cholesterol quantification, it turned out that silencing of YY1 attenuated the inflammatory response and lipid accumulation in RAW264.7 cells caused by ox-LDL. Moreover, results from the JASPAR database, chromatin immunoprecipitation (ChIP) assay, luciferase reporter assay and Western blot analysis suggested that YY1 activated PCSK9 by binding to PCSK9 promoter and modulated the expression of LDLR in the downstream of PCSK9. In addition, the results of functional experiments demonstrated that the inhibitory effects of YY1 interference on ox-LDL-mediated macrophage inflammation and lipid accumulation were reversed by PCSK9 overexpression. To sum up, YY1 depletion inhibited its activation of PCSK9, thereby reducing cellular inflammatory response, cholesterol homeostasis imbalance, and lipid accumulation caused by ox-LDL.

## Introduction

Dyslipidemia plays an important role in the process of atherosclerosis and induces a variety of cardiovascular events, including myocardial infarction, stroke, and heart disease [1]. During this process, macrophages maintain low-density lipoprotein (LDL) and cholesterol homeostasis in peripheral blood [2]. When LDL is oxidized, scavenger receptors (SR), especially lectin-like, oxidized low-density lipoprotein receptor-1 (LOX-1), which functions in a variety of cells including macrophages to increase inflammation and lipid accumulation as well as to stimulate the formation of foam cells, are upregulated [3, 4]. Despite a variety of LOX-1-targeted therapies being clinically available for atherosclerosis, such as RNA interference and targeted monoclonal antibodies to stimulate foam cell formation [3, 4], the underlying molecular mechanism remains to be elucidated.

Transcription factor Yin Yang 1 (YY1) is a multifunctional, zinc finger structural protein, ubiquitously expressed and highly conserved among species, with varied roles according to cell type [5, 6]. In liver cells, YY1 is known to be a vital driver in regulating the expressions of lipid metabolism-related genes. By inhibiting fatty acid β-oxidation, YY1 can increase triglyceride level, lipid accumulation, and the probability of hepatocellular carcinoma onset [7, 8]. In oligodendrocytes, YY1 maintains its immature state by suppressing myelin proteolipid protein (Plp1) expression [9]. In addition, in lipopolysaccharide (LPS)-stimulated microglia, YY1 is also found to interact with NF-κB via reducing the H3K27ac modification on the interleukin 6 (*IL-6*) promoter, ultimately increasing *IL-6* transcription and promoting neuroinflammation [10]. In one novel research case, YY1 expression was found to be upregulated in ox-LDL-stimulated macrophages, but whether it can participate in the secretion of macrophage pro-inflammatory factors and lipid accumulation remains elusive and the potential molecular mechanisms also need to be further explored [11].

Proprotein convertase subtilisin/kexin type 9 (PCSK9) is a promising therapeutic target that has been widely studied for the treatment of atherosclerosis [12-15]. PCSK9 has been found to promote the release of cyto-inflammatory factors which are mainly dependent on low-density lipoprotein receptor (LDLR) in macrophages [16]. PCSK9 has also been found to affect cholesterol homeostasis by transferring LDLR to lysosomes for degradation [17]. Moreover, overexpression of PCSK9 may also induce degradation of LDLR in the lumen of the endoplasmic reticulum through a non-proteasome mechanism [18].

Collectively, in this study we explored whether YY1 interference could alleviate ox-LDL-induced macrophage inflammation and lipid accumulation through the PCSK9/LDLR signaling pathway by transcriptional regulation of PCSK9.

## Materials and Methods

### Bioinformatics Analysis

The JASPAR CORE database (http://jaspar.genereg.net/) was used to predict the binding sites of YY1 on the PCSK9 promoter (Site 1, -1724~-1713; Site 2, -662~-651).

### Cell Culture

Mouse mononuclear macrophage RAW264.7 cells purchased from The Cell Bank of Type Culture Collection of The Chinese Academy of Sciences were maintained in Dulbecco’s modified Eagle’s medium (DMEM; Sigma, Germany) containing 10% fetal bovine serum (FBS; Gibco, USA) and 1% penicillin-streptomycin solution (Beyotime Biotechnology, China), and incubated at 37˚C with 5% CO_2_.

### Cell Transfection and Treatment

Short hairpin RNAs (shRNAs) synthesized and purified by Shanghai GenePharma Co., Ltd. were transfected into RAW264.7 cells for specific shRNA-mediated inhibition using Lipofectamine 3000 (Invitrogen, USA) according to the manufacturer’s recommendation. A scrambled shRNA (shRNA-NC) was used as a negative control. The sequences used in this study were as follows: shRNA-YY1-1, sense 5’-CGGCGACGACGACTACATA-3’, antisense 5’-TATGTAGTCGTCGTCGCCG-3’; shRNA-YY1-2, sense 5’-GTTGAGAGCTCAAAGCTAA-3’, antisense 5’-TTAGCTTTGAGCTCTCAAC-3’; shRNA-NC, sense 5’-GATCCC CTTCTCCGAACG-3’, antisense 5’-AGCTAAAAATTCTCCGAAC-3’. Recombinant plasmid pcDNA3.1-PCSK9 (Ov-PCSK9) constructed by YouBio was transfected into RAW264.7 cells using Lipofectamine 3000 for specific PCSK9 overexpression according to the manufacturer’s specification, and the unloaded plasmid pcDNA3.1 (Ov-NC) was used as a negative control. For additional ox-LDL (Kalen Biomedical, USA) treatment, the transfected or untransfected RAW264.7 cells were incubated with 60 μg/ml or 100 μg/ml ox-LDL at 37°C for 48 h.

### Western Blotting Analysis

After the rinse with PBS (Corning, USA), a total of 5 × 10^6^ transfected or untransfected RAW264.7 cells were collected for total protein isolation by adding lysis buffer (SbjBio, China) containing a protease inhibitor cocktail for general use (Beyotime Biotechnology). Then, 20 μg protein lysates was loaded into SDS-PAGE per lane for separation and then transferred onto PVDF membrane (Millipore, USA) for Western blotting. The antibodies used in this study were as follows: YY1 (rabbit, 1:9,000, abcam, cat. #ab245365); PCSK9 (rabbit, 1:1,000, abcam, cat. #ab185194); LDLR (rabbit, 1:800, abcam, cat. #ab52818); *GAPDH* (rabbit, 1:12,000, abcam, cat. #ab181602); NLRP3 (rabbit, 1:1,000, abcam, cat. #ab270449); ASC (rabbit, 1:1,000, Cell Signaling Technology, cat. #67824); caspase-1 (rabbit, 1 μg/ml, abcam, cat. #ab138483); goat anti-rabbit-HRP (1:25,000, Jackson ImmunoResearch, cat. #111-035-003).

### Reverse Transcription‐Quantitative Polymerase Chain Reaction (RT-qPCR)

Total RNA was extracted from 1 × 10^5^ RAW264.7 cells by using TRIzol reagent (Thermo Fisher Scientific, Inc., USA), and then 2 μg of total RNA was reversed into cDNA using the Superscript Kit (Invitrogen) according to the manufacturers’ instructions. Subsequently, RT-qPCR was performed using SybrGreen (Life Technologies, USA) and detected by an ABI PRISM 7700 detection system. The primer sequences used were as follows: YY1, forward 5’-ATGAGAAAGCATCTGCACACC-3’ and reverse 5’-AGCCTTCGAATGTGCACTGAAA-3’; PCSK9, forward 5’-GCGAATTATCCCAGCATGGC-3’ and reverse 5’-CACACTTGCTCGCCTGTCT-3’; LDLR, forward 5’-CGAAGCCATTTTCAGTGCCA-3’ and reverse 5’-TCACACCAGTTCACCCCTCTA-3’; *GAPDH*, forward 5’-AAGAGGGATGCTGCCCTTAC-3’ and reverse 5’-CCAATACGGCCAAATCCGTTC-3’; TNF-α, forward 5’-GTAGCCCACGTCGTAGCAAA-3’ and reverse 5’-ACAAGGTACAACCCATCGGC-3’; *IL-6*, forward 5’-GGGACTGATGCTGGTGACAA-3’ and reverse 5’-TGCCATTGCACAACTCTTTTC-3’; IL-1β, forward 5’-TGCCACCTTTTGACAGTGATG-3’ and reverse 5’-TGATGTGCTGCTGCGAGATT-3’; IL-10, forward 5’-TGAATTCCCTGGGTGAGAAGC-3’ and reverse 5’-AGACACCTTGGTCTTGGAGCTTATT-3’.

### Oil Red O Staining

RAW264.7 cells seeded in 24-well plates (5 × 10^4^/well) were fixed with 4% paraformaldehyde (PFA; Sigma) at room temperature for 15 min and then washed twice with PBS. Subsequently, the Oil Red O staining was performed by using an Oil Red O Staining Kit (Beyotime Biotechnology) according to the manufacturer’s instructions. The images were taken by a light microscope at 200× magnification (Olympus Corporation, Japan).

### Cellular Cholesterol Quantitation Analysis

The Cholesterol Quantitation Kit (Sigma-Aldrich) was used to analyze the total cholesterol (TC), free cholesterol (FC), and cholesteryl ester (CE) in RAW264.7 cells according to the manufacturer’s instructions.

### Chromatin Immunoprecipitation (ChIP) Assay

After treatment with 100 μg/ml ox-LDL at 37°C for 48 h, three confluent 10-cm dishes of RAW264.7 cells were used to perform the ChIP assay in triplicate for each sample using a Chromatin Immunoprecipitation Assay Kit (Beyotime Biotechnology) according to the manufacturer’s instructions.

### Dual Luciferase Gene Reporter Assay

The promoter region of murine PCSK7 containing predicted YY1 binding site 2 (Site 2-WT, GAGGATGGTCCC) and its mutant (Site 2-MUT, CTCCTACCAGGG) was cloned into the pGL2 plasmid and co-transfected with YY1 overexpression plasmid into RAW264.7 cells. The mutant was constructed using the MutanBEST Kit (TaKaRa, Japan) according to the manufacturer’s recommendations. Subsequently, a Dual-Lumi Luciferase Reporter Gene Assay Kit (Beyotime Biotechnology) was used to detect the luciferase activity according to the manufacturer’s instructions.

### Statistical Analysis

The GraphaPad Prism 8 software was used to perform statistical analysis, and the data were shown as the mean± SD. One-way ANOVA followed by Tukey’s post-hoc test was performed to compare the differences among multiple groups. *p*-values less than 0.05 were considered to be statistically significant.

## Results

### Ox-LDL Upregulates YY1 and PCSK9 Expressions but Downregulates LDLR Expression in Macrophages

**Fig. 1:**
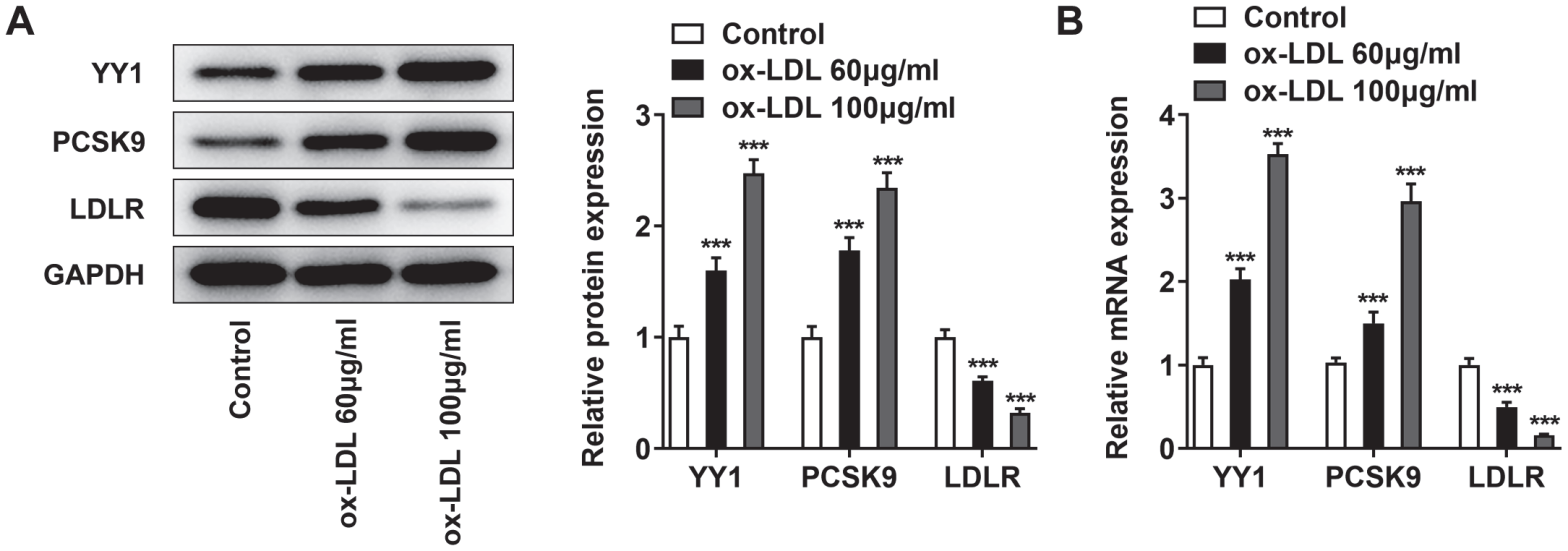
Ox-LDL upregulated YY1 and PCSK9 expressions but downregulated LDLR expression in macrophages. (**A**) RAW264.7 cells were treated with different doses of ox-LDL (60 μg/ml and 100 μg/ml) for 48 h. Western blot analysis of the protein levels of YY1, PCSK9 and LDLR. *GAPDH* was used as the loading control. (**B**) RT-qPCR analysis of the mRNA levels of YY1, PCSK9 and LDLR. The normal untreated RAW264.7 cells were used as the control. *** *p* < 0.001 vs. control group. Ox-LDL, oxidized low-density lipoprotein; RT-qPCR, quantitative real‐time reverse transcription‐polymerase chain reaction; YY1, Yin Yang 1; PCSK9, proprotein convertase subtilisin/kexin type 9; LDLR, low-density lipoprotein receptor; *GAPDH*, glyceraldehyde-3-phosphate dehydrogenase.

Through Western blot and RT-qPCR analysis, after mouse macrophage RAW264.7 cells were treated with 60 μg/ml and 100 μg/ml ox-LDL for 48 h, a significant increase in YY1 and PCSK9 expressions in a dose-dependent manner was noticed compared with that in untreated RAW164.7 cells. Interestingly, the expression of LDLR also showed a marked decrease in a dose-dependent manner. In the highest dose group, the protein level was reduced approximately 2.5-fold, and the mRNA level was reduced about 3.5-fold compared with the untreated group (Figs. 1A and 1B). For further investigation, ox-LDL at a dose of 100 μg/ml was selected for following experiments. Hence, we concluded that ox-LDL treatment upregulated YY1 and PCSK9 expressions but downregulated LDLR expression in RAW264.7 cells.

### Silencing of YY1 Relieves ox-LDL-Induced Inflammatory Response in Macrophages

**Fig. 2:**
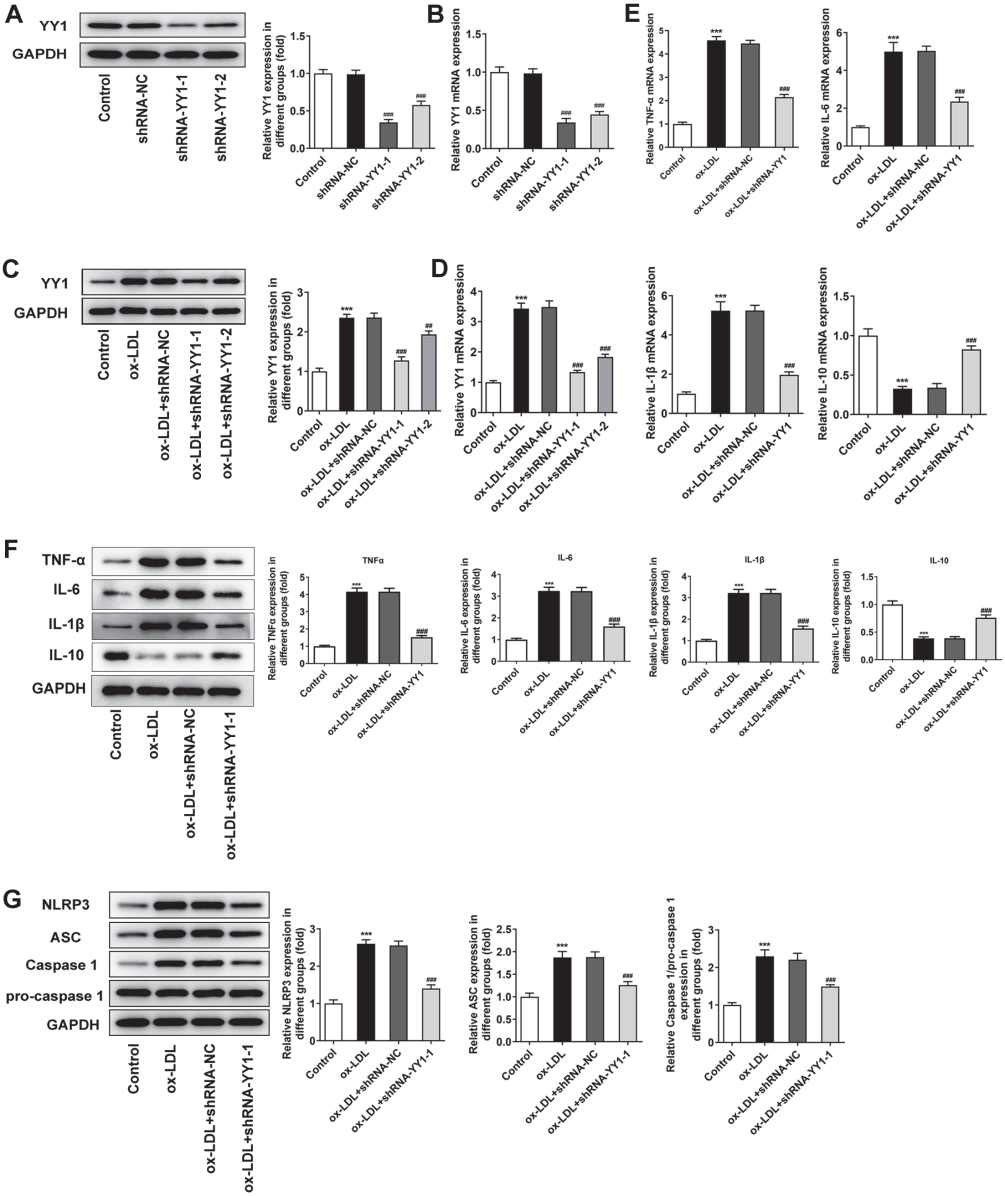
Silencing of YY1 relieved ox-LDL-induced inflammatory response in macrophages. The interference efficiency of YY1 in RAW264.7 cells with the absence (**A, B**) or presence (**C, D**) of 100 μg/ml ox-LDL treatment was examined by Western blot analysis and RT–qPCR analysis. *GAPDH* was used as the loading control. (**E, F**) RT-qPCR analysis and Western blot of TNF-α, *IL-6*, IL-1β and IL-10 levels in RAW264.7 cells treated with 100 μg/ml ox-LDL. (**G**) Western blot analysis of the release of inflammasome in RAW264.7 cells treated with 100 μg/ml ox-LDL. *GAPDH* was used as the loading control. The normal untreated RAW264.7 cells were used as the control. ***p < 0.001 vs. Control group; ##*p* < 0.01, ###*p* < 0.001 vs. shRNA-NC or ox-LDL + shRNA-NC group. Ox-LDL, oxidized low-density lipoprotein; RT-qPCR, quantitative real‐time reverse transcription‐polymerase chain reaction; YY1, Yin Yang 1; *GAPDH*, glyceraldehyde-3-phosphate dehydrogenase; TNF-α, tumor necrosis factor; *IL-6*, interleukin 6; IL-1β, interleukin 1 beta; IL-10, interleukin 10; NLRP3, NLR family pyrin domain containing 3; ASC, PYD and CARD domain containing.

To silence the expression of YY1, specific shRNAs (shRNA-YY1-1 and shRNA-YY1-2) were transfected into RAW264.7 cells. Western blot and RT-qPCR analyses revealed that shRNA-YY1-1 showed better interference efficiency in RAW164.7 cells with (Figs. 2C and 2D) or without (Figs. 2A and 2B) ox-LDL administration. To explore the role of YY1 in ox-LDL-mediated macrophage inflammatory response, the releases of inflammatory factors and inflammasomes were respectively detected. The RT-qPCR analysis and Western blot results demonstrated that ox-LDL treatment led to decreased IL-10 expression and increased expressions of TNF-α, *IL-6*, and IL-1 β, which was consistent with a previous study [19]. Moreover, after YY1 was silenced, the pro-inflammatory effects of ox-LDL treatment were weakened compared with that in ox-LDL + shRNA-NC group (Figs. 2E and 2F). Western blot analysis also showed that YY1 inhibition reduced ox-LDL-mediated inflammasome release, accompanied with the downregulated expressions of receptor protein NLRP3, adaptor protein ASC, and effector protein caspase-1 in ox-LDL-exposed RAW264.7 cells transfected with shRNA-YY1-1 compared with that in ox-LDL-exposed RAW264.7 cells transfected with shRNA-NC (Fig. 2G). These findings indicated that YY1 elimination alleviated ox-LDL-induced inflammatory response in macrophages.

### YY1 Depletion Reduces Ox-LDL-Mediated Lipid Accumulation in Macrophages

**Fig. 3:**
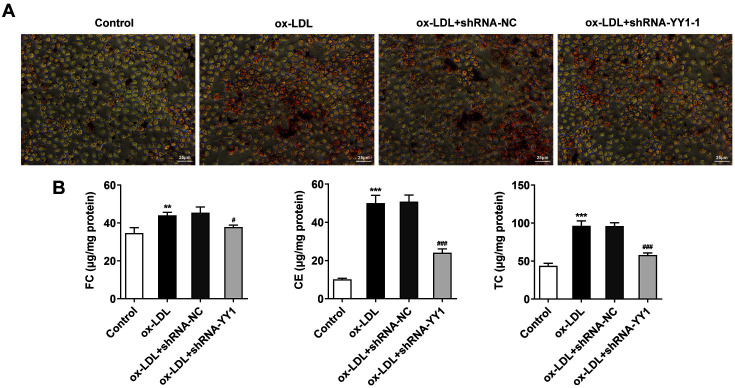
YY1 depletion reduced ox-LDL-mediated lipid accumulation in macrophages. (**A**) Oil Red O staining assay showed lipid accumulation (Lipid drops, red; scale bar, 25 μm) in RAW264.7 cells treated with 100 μg/ml ox-LDL. (**B**) Cellular cholesterol quantitation analysis of FC, CE and TC expression in RAW264.7 cells treated with ox-LDL. **p < 0.01, ***p < 0.001 vs. Control group; #*p* < 0.05, ###*p* < 0.001 vs. ox-LDL + shRNA-NC group. Ox-LDL, oxidized low-density lipoprotein; YY1, Yin Yang 1; TC, total cholesterol; FC, free cholesterol; CE, cholesteryl ester.

To assess the role of YY1 in ox-LDL-mediated macrophage lipid accumulation, Oil Red O staining was implemented. The results showed an obvious lipid accumulation (Lipid drops, Red) in cells with ox-LDL treatment, while YY1 deficiency decreased the accumulation of lipid compared with that in ox-LDL + shRNA-NC (Fig. 3A). To further determine the function of YY1 in intracellular cholesterol homeostasis, the cellular cholesterol content was analyzed. The data showed dramatic increases in FC, CE, TC and CE/TC levels in the ox-LDL group relative to the control group. Also, compared with that in the ox-LDL + shRNA-NC group, when YY1 was downregulated, ox-LDL-mediated cholesterol metabolism disorders were alleviated, with CE/TC below 50%(Fig. 3B). These results suggested that YY1 participated in ox-LDL-mediated lipid and cholesterol metabolism disorders in macrophages.

### YY1 Mediates the Activation of PCSK9 in RAW264.7 Cells

**Fig. 4:**
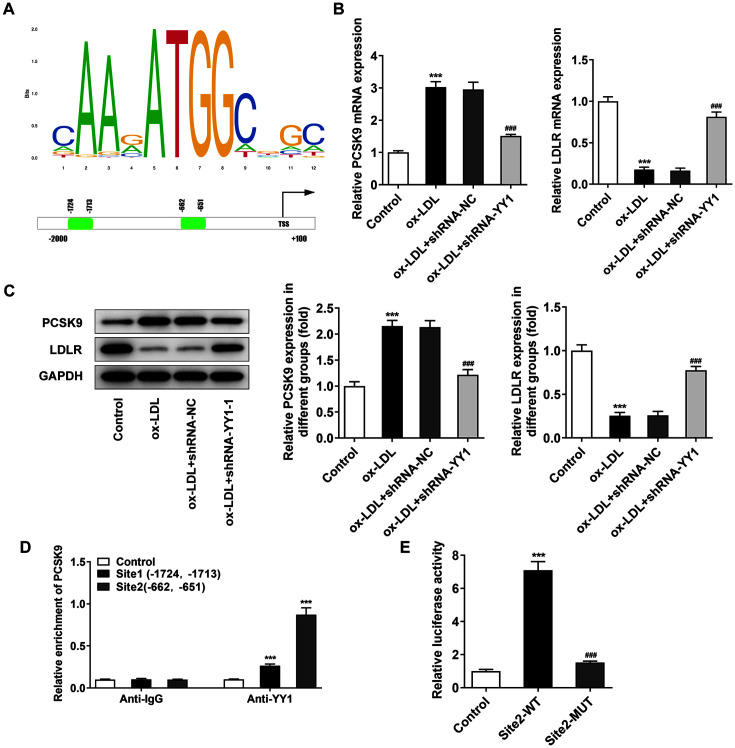
YY1 mediated the activation of PCSK9 in RAW264.7 cells. (**A**) The JASAR CORE database showed the predicted binding motif and sites (Site 1, -1724~-1713; Site 2, -662~-651) of YY1 on the PCSK9 promoter. (**B** and **C**) RT-qPCR analysis and Western blot analysis of PCSK9 and LDLR expressions in ox-LDL-treated RAW264.7 cells. (**D**) ChIP assay showed the binding sites (Site 1, Site 2) of YY1 to the promoter of PCSK9. Anti-IgG served as the control. ***p < 0.001 vs. Anti-IgG group. (**E**) Luciferase reporter assay showed the luciferase activity of PCSK9 site 2-WT and site 2-MUT in RAW264.7 cells. ***p < 0.001 vs. control group; ###*p* < 0.001 vs. Site 2-WT group. *GAPDH* was used as the loading control. The normal untreated RAW264.7 cells were used as the control. ***p < 0.001 vs. Control group; ###*p*<0.001 vs. ox-LDL + shRNA-NC group. YY1, Yin Yang 1; PCSK9, proprotein convertase subtilisin/kexin type 9; LDLR, low density lipoprotein receptor; *GAPDH*, glyceraldehyde-3-phosphate dehydrogenase; Ox-LDL, oxidized low-density lipoprotein; ChIP, chromatin immunoprecipitation.

The promoter region of PCSK9 as well as the obtained two YY1 binding sites (Site 1, -1724~-1713; Site 2, -662~-651) were analyzed through the JASPAR database (Fig. 4A). YY1 elimination was found to partly reverse ox-LDL-mediated PCSK9 upregulation and the subsequent downregulation of LDLR at protein level (Figs. 4B and 4C). To explore the interaction of YY1 and PCSK9, the ChIP assay was performed. PCSK9 promoter was observed to be abundant in YY1 antibody. Moreover, YY1 binding site 2 showed a higher enrichment rate than site 1 (Fig. 4D). Therefore, YY1 might interact with the promoter of PCSK9 at Site 2. The luciferase reporter assay also demonstrated that YY1 overexpression obviously enhanced the luciferase activity at Site2-WT instead of Site2-MUT (Fig. 4E). Taken together, YY1 bound to the promoter region of PCSK9 in ox-LDL-treated macrophages and resulted in the decrease of LDLR.

### PCSK9 Overexpression Inhibits the Release of Inflammatory Response and Lipid Accumulation in Macrophages Induced by Ox-LDL after Blocking YY1 Interference

**Fig. 5:**
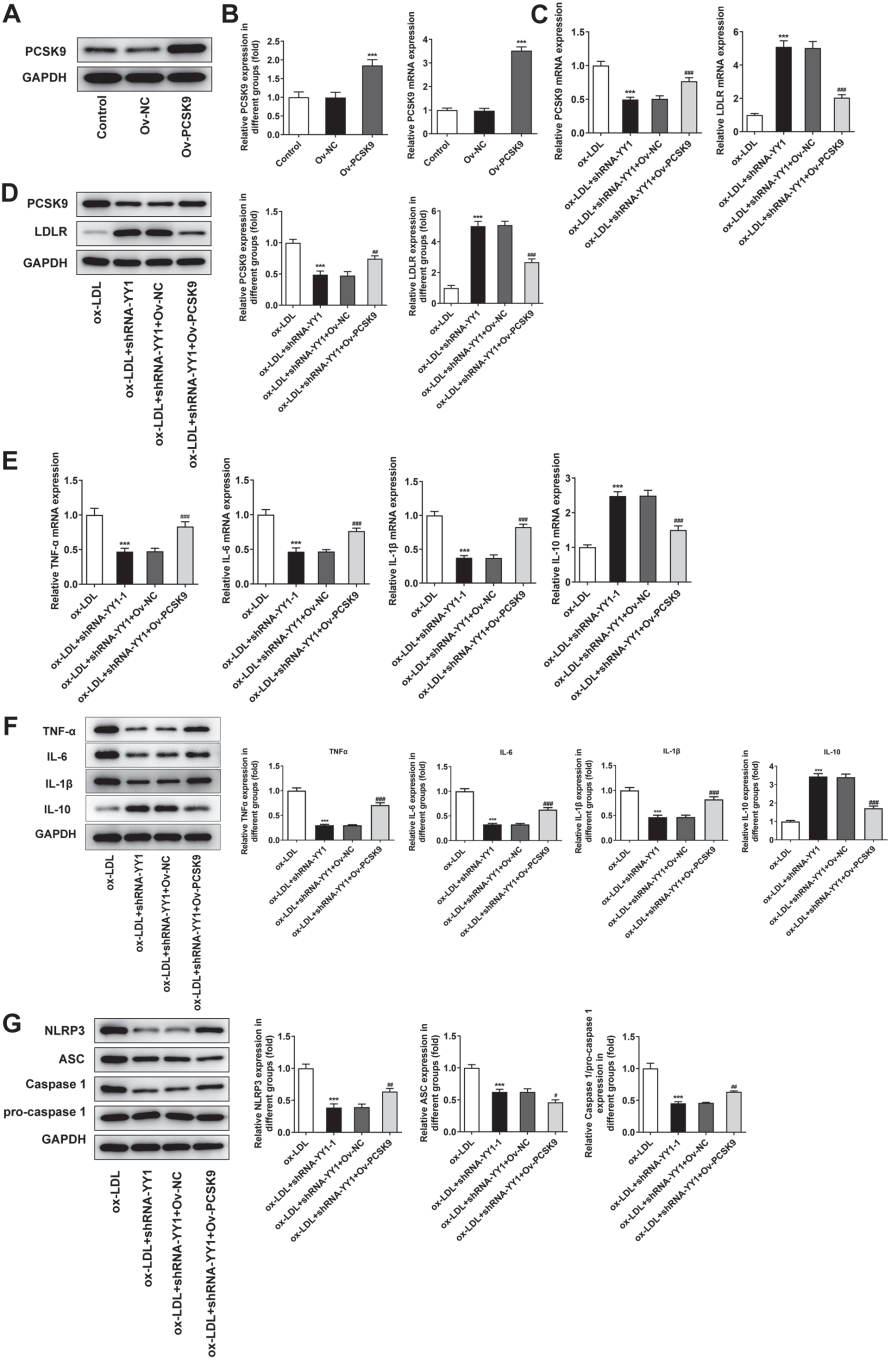
PCSK9 overexpression inhibits the release of inflammatory response in macrophages induced by ox-LDL after blocking YY1 interference. The overexpression efficiency of the recombinant plasmids pcDNA3.1-PCSK9 (Ov-PCSK9) in RAW264.7 cells was tested by (**A, B**) Western blot analysis and (**C**) RT-qPCR analysis. *GAPDH* was used as the loading control. The normal untransfected RAW264.7 cells were used as the control. ***p < 0.001 vs. Ov-NC group. (**D**) Western blot analyzed PCSK9 and LDL protein levels in ox-LDL-treated RAW164.7 cells transfected with shRNA-YY1 and Ov-PCSK9. (**E, F**) RT-qPCR analysis and Western blot of TNF-α, *IL-6*, IL-1β and IL-10 levels in RAW264.7 cells treated with ox-LDL. (**G**) Western blot analysis of inflammasome release in RAW264.7 cells treated with 100 μg/ml ox-LDL. *GAPDH* was used as the loading control. ***p < 0.001 vs. ox-LDL group; #*p* < 0.05, ##*p* < 0.01, ###*p* < 0.001 vs. ox-LDL + shRNA-YY1 + Ov-NC group. YY1, Yin Yang 1; PCSK9, proprotein convertase subtilisin/kexin type 9; LDLR, low-density lipoprotein receptor; *GAPDH*, glyceraldehyde-3-phosphate dehydrogenase; Ox-LDL, oxidized low-density lipoprotein; RT-qPCR, quantitative real‐time reverse transcription‐polymerase chain reaction; TNF-α, tumor necrosis factor; *IL-6*, interleukin 6; IL-1β, interleukin 1 beta; IL-10, interleukin 10; NLRP3, NLR family pyrin domain containing 3; ASC, PYD and CARD domain containing.

**Fig. 6:**
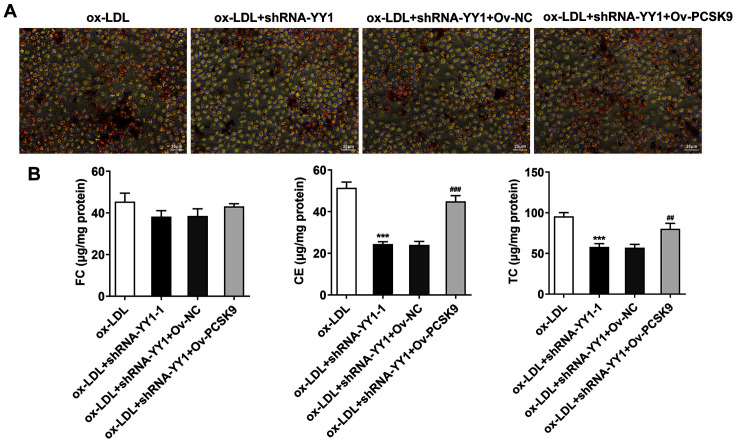
PCSK9 overexpression inhibits the lipid accumulation in macrophages induced by ox-LDL after blocking YY1 interference. (**A**) Oil Red O staining assay showed lipid accumulation (Lipid drops, red; scale bar, 25 μm) in RAW264.7 cells treated with 100 μg/ml ox-LDL. (**B**) Cellular cholesterol quantitation analysis of FC, CE and TC expressions in ox-LDL-treated RAW264.7 cells. ***p < 0.001 vs. ox-LDL group; ##*p* < 0.01, ###*p* < 0.001 vs. ox-LDL + shRNA-YY1 + Ov-NC group. Ox-LDL, oxidized low-density lipoprotein; YY1, Yin Yang 1; PCSK9, proprotein convertase subtilisin/kexin type 9; TC, total cholesterol; FC, free cholesterol; CE, cholesteryl ester.

Based on the findings above, it was hypothesized that LDLR was a downstream gene of PCSK9. Then, the recombinant plasmid pcDNA3.1-PCSK9 (Ov-PCSK9) was constructed and transfected into RAW264.7 cells to overexpress PCSK9. The plasmids showed a successful overexpression transfection at both mRNA and (~ 3.5-fold increase) and protein levels (~ 2-fold increase) when compared with the Ov-NC group (Figs. 5A-5C). Furthermore, the dramatic decrease of LDLR imposed by PCSK9 overexpression was noticed, although YY1 inhibition upregulated its expression (Fig. 5D). Following that, the function of PCSK9 in ox-LDL-mediated macrophage inflammatory response, and the release of inflammatory factors and inflammasomes were detected respectively. RT-qPCR analysis and Western blot showed that PCSK9 overexpression remarkably reversed the altered expressions of cyto-inflammatory factors induced by YY1 deficiency in ox-LDL-treated RAW264.7 cells (Figs. 5E and 5F). Additionally, Western blot analysis also showed similar results in the release of inflammasomes (Fig. 5G). Subsequently, results obtained from Oil Red O staining and cholesterol content detection showed that YY1 silencing alleviated the abnormal lipid and cholesterol metabolism of macrophages, which were countervailed due to the overexpression of PCSK9 (Figs. 6A and 6B). Taken together, these data indicated that YY1 induced by ox-LDL treatment activated PCSK9 in RAW264.7 cells, resulting in the degradation of LDLR at protein level, which ultimately led to intracellular inflammatory response as well as lipid and cholesterol metabolism disorders.

## Discussion

Foam cells formed by macrophages have been shown to play a crucial role in the pathogenesis of atherosclerosis [20, 21]. A previous study demonstrated a notable increase of YY1 expression in a dose-dependent manner in ox-LDL-stimulated macrophages [11]. We have confirmed that ox-LDL stimulation led to increased YY1 expression in mouse macrophage RAW264.7 cells. Furthermore, our data also revealed high expression of PCSK9 and low expression of LDLR in ox-LDL-insulted RAW264.7 cells. Numerous studies have confirmed that PCSK9 is a serine protease which promotes the process of atherosclerosis by actively targeting and causing the degradation of LDLR [13, 14, 22, 23]. In human HuH7v hepatocarcinoma cells, LDLR mRNA is increased by βE2-conditioned medium with PCSK9 [24]. Epigallocatechingallate decreased the mRNA levels of PCSK9 and increased LDLR mRNA expression in hepatic cells [25]. However, the specific function of YY1 in ox-LDL-treated macrophages has not been elucidated in previous studies.

Considering that a strong correlation between ox-LDL exposure and YY1 overexpression has been confirmed, the possible role of YY1 in this situation was further explored. It is well known that ox-LDL promotes the formation and progression of atherosclerosis by increasing intraplaque release of inflammatory cytokines in macrophages through several SRs [4]. YY1 has been shown to promote neuroinflammation through NF-κB-mediated activation of *IL-6* expression in LPS-stimulated microglia [10]. Additionally, in the present study, we found that YY1 inhibition alleviated the release of macrophage pro-inflammatory factors, including TNF-α, *IL-6*, and IL-1β caused by ox-LDL. Recent studies have shown that nucleotide-binding oligomerization domain-like receptor protein 3 (NLRP3) inflammasomes, which are activated by ox-LDL, aggravate atherosclerosis through caspase-1-induced IL-1β secretion [26, 27]. Our results suggested that YY1 can promote ox-LDL-mediated macrophage pro-inflammatory phenotypic transition through the release of NLRP3 inflammasomes. Recently, the Dixit VD team found that the release of NLRP3 inflammasomes in adipose tissue macrophages causes lipid metabolism disorders through catechol degradation [28].

YY1 is a negative regulator of gene transcription in response to multiple sterol regulatory element-binding proteins [5]. Various studies have demonstrated that YY1 is closely related to lipid metabolism disorders [6]. Abnormal YY1 expression provides the driving force for tumor development by inhibiting fatty acid oxidation and promoting lipid accumulation [8]. In the current study, we further determined the effects of ox-LDL-mediated YY1 upregulation on abnormal lipid metabolism. Lipids are internalized and transported to lysosomes through phagocytosis, and are digested by lysosomal acid lipase to release free cholesterol. Free cholesterol is converted into cholesterol ester and stored in the endoplasmic reticulum by acetyl-CoA acetyltransferase treatment. After treatment with neutral cholesterol hydrolase, free cholesterol is formed again, which constitutes cholesterol circulation [2]. Our results revealed that YY1 elimination reduced the number of red-stained particles in macrophages after ox-LDL stimulation when the cell cholesterol ester content was less than 50% of the total cholesterol. These findings indicated that YY1 elevation in ox-LDL-mediated macrophages disrupted the maintenance of cholesterol homeostasis and promoted lipid accumulation.

PCSK9, which is involved in the dysregulation of cholesterol metabolism mainly through the degradation of LDLR, promotes the formation of foam cells by releasing inflammatory mediators in the process of atherosclerosis [22]. A trial involving 27,564 patients with atherosclerotic cardiovascular disease showed that the use of Evolocumab, a monoclonal antibody that inhibits PCSK9, reduced LDL cholesterol levels by about 60% [29]. PCSK9 directly enhanced the progression of atherosclerotic lesions via promoting vascular inflammation [30-32]. The plasma PCSK9 amounts were positively related to the white blood cell counts, indicating the presence of chronic low‐grade inflammation and its subset of lymphocytes in stable coronary artery disease patients [33, 34]. PCSK9 plays an important role in inflammation and serves as an inflammatory mediator in atherosclerosis [35]. LPS administration decreased the levels of TNF‐α, IL‐6, IL‐10, MCP‐1, and MIP‐2 [36] but increased IL‐1β level in PCSK9^−/−^ mice [37]. Another study in knockout mice has shown that NLRP3 inflammasomes induce PCSK9 secretion through IL-1β [27]. Combined with the aforementioned experimental results that YY1 promoted the release of NLRP3 inflammasomes in ox-LDL-stimulated macrophages, it could be speculated that there was a connection between YY1 and PCSK9. YY1 decreased TNFα, *IL-6*, and IL-1β levels and increased IL‐10 level in ox-LDL-treated RAW264.7 cells, which were subsequently reversed by PCSK9 overexpression, indicating that YY1 affected inflammation through PCSK9-LDLR pathway. The analysis results of the JASPAR database confirmed the rationality of our conjecture, and the results showed that the YY1 binding motif existed in the promoter region of PCSK9. However, the mechanism under the regulation of YY1 on target genes is very complicated and varies by cell type. YY1 can be used as a activator, repressor, or initiation element-binding protein [9]. We therefore conducted ChIP and luciferase reporter assays and found that in ox-LDL-treated RAW264.7 cells, YY1 activated the expression of PCSK9 by binding to the promoter region of PCSK9, causing subsequent degradation of LDLR at protein level. Finally, we found that PCSK9 was overexpressed in RAW264.7 cells and the lipid accumulation in cells that was previously reduced due to interference of YY1 was increased again. During this process, the release of NLRP3 inflammasomes and inflammatory factors from macrophages was also intensified. These results further confirmed that YY1 played an important role in ox-LDL-mediated macrophage inflammation and lipid accumulation through PCSK9/LDLR signaling.

Our current research only reveals that YY1 promotes the release of macrophage inflammatory factors, abnormal cholesterol metabolism, and lipid accumulation through PCSK9/LDLR signaling. However, the YY1-mediated regulatory mechanism is quite complicated and may also involve histone modification. The existence of other molecular mechanisms remains to be further explored. In addition, the specific mechanism by which YY1 promotes the pro-inflammatory phenotype transformation of macrophages requires in-depth investigation. In the future, human cells and animal models will need to be used to verify a conclusion.

The expression of YY1 showed a dose-dependent increase in ox-LDL-treated RAW264.7 cells. Elevation of YY1 promoted the release of cellular NLRP3 inflammatory bodies and inflammatory factors by activating PCSK9 expression, causing imbalance of cholesterol homeostasis and accumulation of cellular lipids. These findings might help us to further understand the underlying molecular mechanism of the formation of macrophages to foam cells and provide novel insights for atherosclerosis therapy.
